# High prevalence of methicillin resistant *Staphylococcus aureus *in the surgical units of Mulago hospital in Kampala, Uganda

**DOI:** 10.1186/1756-0500-4-326

**Published:** 2011-09-07

**Authors:** David P Kateete, Sylvia Namazzi, Moses Okee, Alfred Okeng, Hannington Baluku, Nathan L Musisi, Fred A Katabazi, Moses L Joloba, Robert Ssentongo, Florence C Najjuka

**Affiliations:** 1 Department of Medical Microbiology, School of Biomedical Sciences, Makerere University College of Health Sciences, Kampala, Uganda; 2 Department of Veterinary Parasitology and Microbiology, School of Veterinary Medicine, Makerere University, Kampala, Uganda; 3 Division of Plastic Surgery, Mulago National Referral and Teaching Hospital, Kampala, Uganda

## Abstract

**Background:**

There is limited data on Methicillin resistant *Staphylococcus aureus *(MRSA) in Uganda where, as in most low income countries, the routine use of chromogenic agar for MRSA detection is not affordable. We aimed to determine MRSA prevalence among patients, healthcare workers (HCW) and the environment in the burns units at Mulago hospital, and compare the performance of CHROMagar with oxacillin for detection of MRSA.

**Results:**

One hundred samples (from 25 patients; 36 HCW; and 39 from the environment, one sample per person/item) were cultured for the isolation of *Staphylococcus aureus*. Forty one *S. aureus *isolates were recovered from 13 patients, 13 HCW and 15 from the environment, all of which were oxacillin resistant and *mecA/femA/nuc*-positive. MRSA prevalence was 46% (41/89) among patients, HCW and the environment, and 100% (41/41) among the isolates. For CHROMagar, MRSA prevalence was 29% (26/89) among patients, HCW and the environment, and 63% (26/41) among the isolates. There was high prevalence of multidrug resistant isolates, which concomitantly possessed virulence and antimicrobial resistance determinants, notably biofilms, hemolysins, toxin and *ica *genes. One isolate positive for all determinants possessed the *bhp *homologue which encodes the biofilm associated protein (BAP), a rare finding in human isolates. SCC*mec *type I was the most common at 54% prevalence (22/41), followed by *SCCmec *type V (15%, 6/41) and *SCCmec *type IV (7%, 3/41). *SCCmec *types II and III were not detected and 10 isolates (24%) were non-typeable.

**Conclusions:**

Hyper-virulent methicillin resistant *Staphylococcus aureus *is prevalent in the burns unit of Mulago hospital.

## Background

There is a global outbreak of Methicillin resistant *Staphylococcus aureus *(MRSA) infections, particularly in industrialized countries [1]. However, there is limited data on MRSA prevalence in Uganda, where ~10% of the surgical procedures become septic with *S. aureus *being the most frequent pathogen isolated [2-4]. Ojulong et al. 2009 determined the prevalence of MRSA in patients with post-operative surgical wound infections in the surgical wards of Mulago hospital in Kampala, Uganda [5], but the distribution of isolates among healthcare workers (HCW) and the environment was not determined. Here, we aimed to determine MRSA prevalence among patients, HCW and the environment in the burns units at Mulago hospital, and compare the performance of CHROMagar with oxacillin for detection of MRSA (since the routine use of chromogenic agar is usually not affordable in most low income settings).

## Methods

### Study setting and sampling

This cross-sectional study was performed during November, 2009 and February, 2010 in the two surgical units specializing in the treatment of patients with burns at Mulago national referral hospital in Kampala, Uganda. Ethical approval was obtained from the Department of Surgery, Mulago Hospital, and the Research and Ethics Committee of Makerere University College of Health Sciences. Informed consent was obtained from patients and HCW. Patients and HCW enrolled had stayed in the hospital units for at least 72 hours. HCW included doctors, nurses and nursing aids that had direct involvement with patients. For the environment, surfaces of frequently handled items (door handles, sinks, surgical trays, beds and table surfaces) were swabbed, as well as air samples on settle plates (with blood agar). For patients, wounds or nostrils were swabbed while for HCW, hands or nostrils were swabbed. One hundred samples (one sample per person or item) from 25 patients, 36 HCW and 39 items (including air settle plates) were obtained using sterile swabs and transported to the laboratory for culture in tubes containing Amies transport medium (Biolab, Budapest, Hungary).

### Cultures and drug susceptibility testing (DST)

Swabs were cultured on blood agar and Mannitol salt agar at 37°C for 24 hours, ensuring growth of distinct colonies. The predominant colony type per sample was selected, and *S. aureus *was identified to species level microbiologically as previously described [6]. Drug susceptibility testing (erythromycin, 15 μg; vancomycin, 30 μg; gentamicin, 10 μg; oxacillin, 1 μg; tetracycline, 30 μg; chloramphenicol, 30 μg; penicillin, 10 units; and sulphamethoxazole-trimethoprim, 1.25/23.15 μg) was performed with the disc diffusion method (Biolab inc, Budapest, Hungary). Isolates resistant to erythromycin were screened for the macrolide-lincosamide and streptrogramin B (MLS_B_) phenotype. MRSA was identified based on oxacillin resistance following standard procedures [7], and confirmed by PCR detection of the *mecA *gene. For comparison, oxacillin resistant-*mecA *positive isolates were further screened for growth on CHROMagar (BD diagnostics, Sparks, USA).

### *SCCmec *genotyping

Staphylococcus Cassette Chromosomal *mec *(*SCCmec*) genotyping was performed in a multiplex PCR protocol described by Boye et al, 2007 (see additional file 1 for primers and PCR conditions) [8]. The SCC*mec *types were determined based on the banding patterns upon agarose gel electrophoresis of the amplicons [8]. Isolates with no visible bands were classified as non-typeable [8].

### Detection of virulence determinants

#### Biofilms

Biofilms were detected with the microtiter plate method [9] and the biofilm unit calculated according to Amaral et al. [10]. Briefly, assays were performed in triplicates in tryptic soya broth (TSB) with 1% glucose in 96-well polystyrene flat-bottom tissue culture plates. Isolates were incubated at 37°C overnight with gentle shaking and standardized to OD_600 _= 0.005 with normal saline. Then, 50 μl of standardized cells mixed with 150 μl TSB/1% glucose were incubated at 37°C for 17 hr. After washing three times with sterile water and staining with crystal violet for 15 minutes, cells were washed again and incubated at room temperature for 1 hr in 95% ethanol. Then, biofilms were measured with a spectrophotometer at OD_570_. The biofilm unit (BU) was calculated using negative control values with the formula A_1_/A_2_, where A_1 _is the test value while A_2 _is the negative control value. Isolates with BU > 2× the negative control value were considered biofilm producers and were classified as: weak, 0.182 < BU < 0.364; moderate, 0.364 < BU < 0.728; strong, BU > 0.728 [10]. The biofilm forming *S. epidermidis *RP62A and its non-biofilm forming variant (ATCC 12228) were used as controls.

#### Virulence and antimicrobial resistance genes

Molecular assays for the detection of virulence associated genes, *ica *(intercellular adhesion); *cna *(collagen adhesion [11]); hemolysins (*hla*, *hlb*, *hld*, *hlg *[12]); *sdrE *(serine-aspartate repeat protein E [11]); *PVL *(Panton-Valentine leukocidin [13]); and *S. aureus *super-antigenic toxins (*tst*, toxic shock syndrome toxin [12,14] and *sea*, staphylococcal enterotoxin A [12,14]) were performed with primers and conditions described in literature (also see additional file 1). To detect genes encoding aminoglycoside-modifying enzymes (AMEs) [15], PCR on *aac(6')-Ie-aph(2'')-Ia *(bifunctional aminoglycoside-6-N-acetyltransferase/2*''*-O- phosphoryltransferase), *aph(3')-IIIa *(aminoglycoside-3'-O-phosphoryltransferase III) and *ant(4')-Ia *(aminoglycoside-4'-O-nucleotidyltransferase I) was performed. Presence of *mecA *(the molecular determinant of methicillin resistance), *vanA*/*vanB1 *(encode vancomycin resistance variants) and *blaZ *(encodes β-lactamase) was also determined by PCR. For *mecA *genotyping, methicillin resistant *S. aureus *(MRSA-252) and methicillin sensitive *S. aureus *(MSSA, ATCC 29213) were used as positive and negative controls, respectively. For analysis, amplicons were electrophoresed on 1% agarose gels in TBE (Tris borate and EDTA) and representative samples sequence-confirmed. The data was analyzed with GraphPad Prism 5 software and presented graphically. Primers and PCR conditions are described in additional file 1. To minimize cross-contamination, DNA extraction and PCR-amplifications were performed in molecular laboratories that are separate from the clinical microbiology laboratory where cultures were grown. The PCR laboratory has designated sections for pre-amplification, amplification and post-amplification.

## Results

Gram positive isolates grew from 62 samples but 11 were lost (five from HCW, four from patients and two from the environment), leaving 51 samples that were completely processed. From these, *S. aureus *grew from 41 samples: 14 from patients (67%, 14/21; 4 from nostrils and 10 from wounds), 13 from HCW (42%, 13/31; 5 from nostrils and 8 from finger swabs), and 14 from the environment (38%, 14/37, see Table 1). Each isolate grew from a distinct sample per person or item. Thus, the prevalence of *S. aureus *in the burns unit was 46% (41/89), (Table 1).

**Table 1:** MRSA prevalence (%) and SCC*mec *types among isolates from the burns units at Mulago hospital

			Oxacillin disc		CHROMagar		SCC*mec *types					
**Sample source (n = 89)**	***S. aureus*^1^**	***mecA ***	**MSSA**	**MRSA**	**MSSA**	**MRSA**	**I**	**II**	**III**	**IV**	**V**	**NT**

Patients (n = 21)	14	14	ND	14	4	10	8	ND	ND	3	1	2

HCW (n = 31)	13	13	ND	13	4	9	8	ND	ND	ND	3	2

Environment (n = 37)	14	14	ND	14	7	7	6	ND	ND	ND	2	6

**Prevalence**	**46** **(41/89)**	**100** **(41/41)**		**46** **(41/89)**	**17** **(15/89)**	**29** **(26/89)**	**54** **(22/41)**	**-**	**-**	**7** **(3/41)**	**15** **(6/41)**	**24** **(10/41)**

^1^Isolates per sample; ND, Not detected; HCW, healthcare worker; NT, Not typeable

All the 41 isolates were sensitive to vancomycin; conversely, all the isolates were oxacillin resistant and *mecA/nuc/femA*-positive (implying they were *S. aureus*), revealing an MRSA prevalence of 46% (41/89) in the burns unit (i.e., among patients, HCW and environment) and 100% (41/41) among isolates (see table 1). SCC*mec *type I was the most common at 54% prevalence (22/41), followed by *SCCmec *type V (15%, 6/41) and *SCCmec *type IV (7%, 3/41). *SCCmec *types II and III were not detected and 10 isolates (24%) were non-typeable (see table 1). For CHROMagar, only 26 isolates grew with the characteristic mauve color indicative of MRSA, revealing a prevalence of 29% in the unit (i.e., among patients, HCW and environment) and 63% (26/41) among isolates.

**Figure 1:**
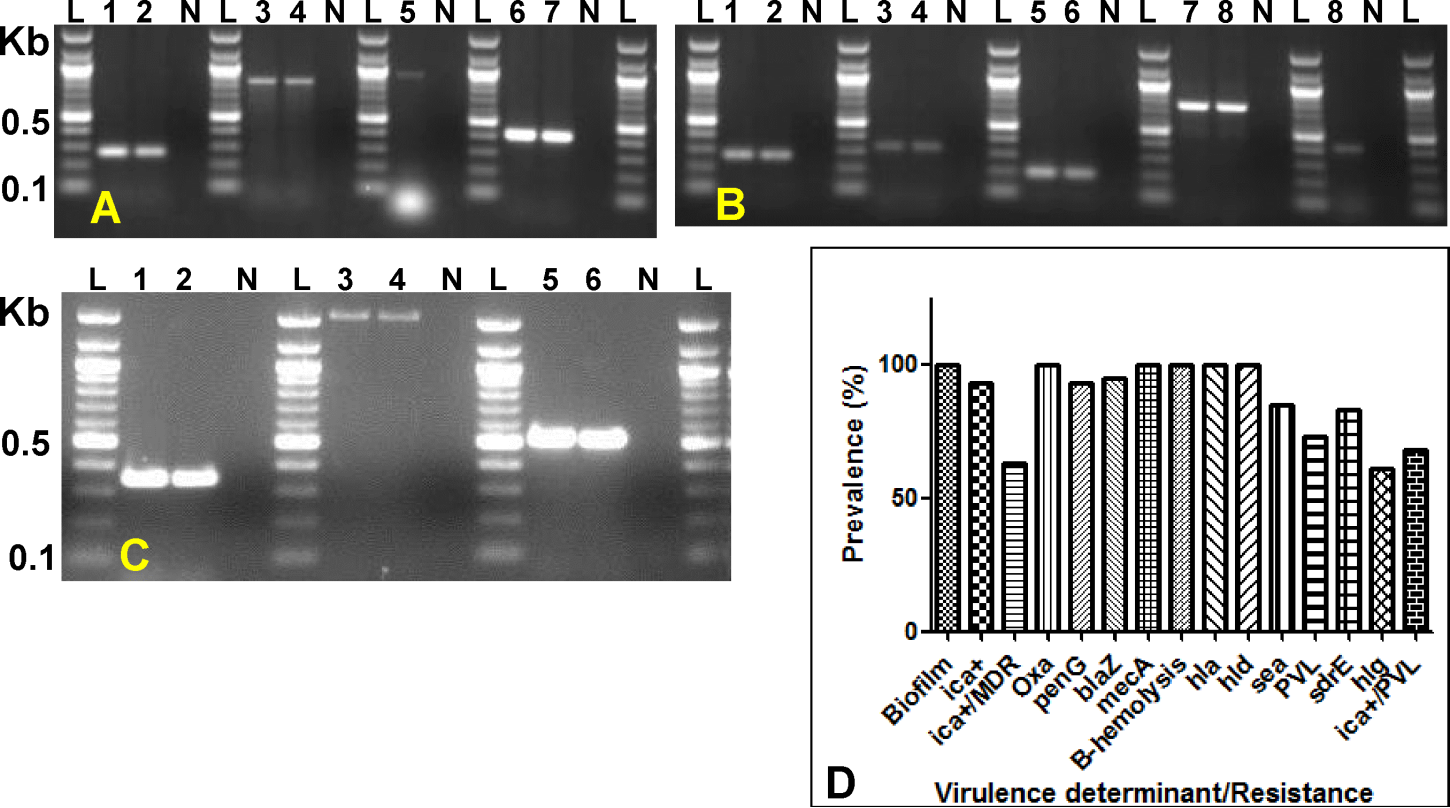
**Representative images showing detection of virulence and antimicrobial resistance genes/determinants in MRSA isolates**. Lanes: L, 100 bp DNA ladder; N, negative control (for all images). **Panel A: **1 & 2, species confirmation of *S. aureus *by detection of the *nuc *gene; 3 & 4, *ica*; 5, bhp; 6 & 7, *PVL*. **Panel B: **1 & 2, *nuc*; 3 & 4, *tst*1; 5 & 6, *hla*; 7 & 8, *sdrE*; 8, *sea*. **Panel C: **Detection of genes encoding aminoglycoside modifying enzymes (AMEs). 1 & 2, (*aac(6')-Ie-aph(2'')-Ia*); 3 & 4, *(aph(3')-IIIa*); 5 & 6, *(ant(4')-Ia*). **Panel B**. Graphical presentation of prevalence for selected determinants.

Overall, 26 six isolates (63%, 26/41) MDR with the commonest pattern being resistance to β-lactams, sulphamethoxazole-trimethoprim (SXT) and tetracycline. Indeed, resistance to penicillin, tetracycline and SXT was high (93%, 38/41; 68%, 28/41 and 66%, 27/41 prevalence, respectively); resistance to penicillin correlated with the high prevalence of *blaZ *gene (93%, 38/41, see Figure 1). Conversely, erythromycin, gentamicin and chloramphenicol resistance was relatively low (29%, 12/41; 20%, 8/41 and 20%, 8/41 prevalence respectively) and correlated with that of the AMEs encoding genes: *aac(6')-Ie-aph(2'')-Ia *(61%, 25/41); *aph(3')-IIIa *(34%, 14/41); and *ant(4')-Ia *(20%, 10/20), which are associated with aminoglycoside resistance [16]. With the exception of one isolate, all the isolates possessing AMEs encoding genes were concomitantly *ica*-positive. Additionally, three isolates resistant to erythromycin gave a positive D-test indicating possible cross-resistance to macrolide-lincosamide-streptogramin (MLS_B_) antibiotics in this setting [17].

Most isolates were strongly biofilm-positive (100%, 41/41, see Figure 1) and nearly all had the *ica *genes (93%, 38/41, with only three testing negative); only one of the two *ica*-negative isolates was MDR. Furthermore, all the isolates exhibited β-hemolysis on blood agar (100%, 41/41) and this correlated with the high prevalence of the genes encoding hemolysins: *hla*, 100% (41/41); *hld*, 100% (41/41); and *hlg*, 61% (25/41)). Many isolates possessed additional virulence genes: *sea*, 85% (35/41); *sdrE*, 83% (34/41); and *PVL*, 73% (30/41), while *cna *and *tsst *were less prevalent (29%, 12/41 and 10%, 4/41 respectively). Thirty four (83%, 34/41) isolates were *ica/sdrE-*positive; 33 (80%, 33/41) *ica*/*sea-*positive; 28 (68%, 28/41) *ica*/*PVL*-positive; 23 (56%, 23/41) *ica*/*hlg*-positive and three (7%, 3/41) *ica/tsst*-positive. All the 12 (29%, 12/41) *cna*-positive isolates were also *ica*-positive. Interestingly, an isolate positive for all the determinants studied possessed the homologue of the *bhp *gene, which encodes the biofilm associated protein (BAP); this is rarely detected in human isolates but frequently detected in staphylococci causing bovine mastitis [18]. This *bhp*-positive isolate was MDR and negative for only three determinants, *tst*, *aph(3')-IIIa *and *ant(4')-Ia*, conforming to its susceptibility to erythromycin/gentamicin. Detailed analyses for the virulence and antimicrobial resistance determinants are provided in additional file 2.

## Discussion

In this study, high prevalence of MDR-MRSA was found in the burns unit of Mulago hospital, predisposing patients to infection with intractable isolates and underscoring the need for improved infection control practices in this setting. Ojulong et al 2009, reported a relatively lower prevalence (31.6%) from the general surgery ward, possibly because this earlier study determined MRSA infections in only patients with post-operative surgical wound infections [5]. Although data are still limited, there are emerging reports of prevalent MRSA infections in sub-Saharan Africa [19].

CHROMagar, generally considered more efficient at detecting MRSA than oxacillin discs [20] was inefficient in this setting (i.e. 46% vs. 29% prevalence, respectively). Since all isolates were oxacillin resistant, *mecA*-positive (*mecA *encodes the penicillin binding protein 2a, the molecular determinant for methicillin resistance [21]), *nuc- *and *femA-*positive, they were confirmed as MRSA. Prior to use, the CHROMagar batch passed quality control screening with known MRSA and MSSA strains; yet only 26 isolates grew mauve colonies (which is indicative of MRSA); the use CHROMagar in this setting may need further investigations.

A high prevalence of biofilm/*ica*-positive isolates correlated with that of MDR isolates. Although biofilms/*ica *genes are debatable as virulence markers [22], in this study, the biofilm/*ica-*positive isolates were concomitantly positive for other virulence genes. Notably was the absolute prevalence for staphylococcal hemolysins: *hla *and *hld*. The *hla *gene encodes a dermanecrotic and neurotoxic toxin that is also responsible for abscess formation; *hld *producing *S. aureus *can cause severe enteritis, while *hlg *lyses mammalian red blood cells and together with *tst*-1 (toxic shock syndrome toxin-1), can be involved in the pathogenesis of toxic shock syndrome (TSS) [15]. *PVL*, also prevalent in this study, causes severe disease in children and young adults with no known exposure to healthcare establishment, and is used as a stable marker for community acquired MRSA [15]. Furthermore, the staphylococcal super-antigenic toxins, *sea *and *tst-1 *were also detected (85% and 10% prevalence respectively). *sea *producing strains are responsible for staphylococcal food intoxications, while *tst *strains produce antigens that are responsible for TSS.

## Conclusion

Hypervirulent methicillin resistant *S. aureus *is prevalent in the burns unit of Mulago hospital.

## Abbreviations

*SCCmec*: Staphylococcus Cassette Chromosomal *mec*; oxa: Oxacillin; penG: Penicillin G; SXT: Sulphamethoxazole-trimethopri; MDR: Multi Drug Resistant

## Competing interests

The authors declare that they have no competing interests.

## Authors' contributions

DPK, SN, MO, AO and FCN performed the molecular genetic studies. MLN, RS, HB and MLJ participated in the design of the study. FCN conceived the study, and participated in its design and coordination. DPK and FCN wrote the manuscript. All authors read and approved the final manuscript.

## Supplementary Material

Additional file 1 **Primers and PCR conditions**. Click here for file

Additional file 2 **Speciation, drug susceptibility testing and detection of virulence and antimicrobial resistance genes/determinants**. NA, Not applicable; ND, Not detected; MSSA, Methicillin Sensitive *Staphylococcus aureus; *MRSE, Methicillin Resistance *Staphylococcus epidermidis*. Click here for file
