# Supplementary material for: High prevalence of methicillin resistant Staphylococcus aureus in the surgical units of Mulago hospital in Kampala, Uganda
Source: BMC Res Notes. 2011 Sep 7;4:326. doi: 10.1186/1756-0500-4-326 (PMC3184088; doi:10.1186/1756-0500-4-326)
Supplement: Additional file 1 — Primers and PCR conditions. [file 1756-0500-4-326-S1.DOC]

**Primers and PCR conditions**

| **Category** | **Name** | **Sequence (5'→3')** | **Target (bp)** | **Reference/**  **Condition^1^** |
| --- | --- | --- | --- | --- |
| Speciation | 16Sx | GGAATTCAAAKGAATTGACGGGGGC (fwd) | 16sRNA (479) | [1] |
|  | 16Sy | CGGGATCCCAGGCCCGGGAACGTATTCAC (rev) |  |  |
|  | nuc1 | GCGATTGATGGTGATACGGTT (fwd) | *nuc* (267) | [2] |
|  | nuc2 | AGCCAAGCCTTGACGAACTAAAGC (rev) |  |  |
|  | femA-2F | AACTGTTGGCCACTATGAGT (fwd) | *femA* (306) | [3] |
|  | femA-2R | CCAGCATTACCTGTAATCTCG (rev) |  |  |
| *SCCmec* typing | Β | ATTGCCTTGATAATAGCCYTCT (fwd) | *ccrA2-B* (937) | [4] |
|  | α3 | TAAAGGCATCAATGCACAAACACT (rev) |  |  |
|  | ccrCF | CGTCTATTACAAGATGTTAAGGATAAT (fwd) | *ccrC* (518) |  |
|  | ccrCR | CCTTTATAGACTGGATTATTCAAAATAT (rev) |  |  |
|  | 1272F1 | GCCACTCATAACATATGGAA (fwd) | *IS*1272 (415) |  |
|  | 1272R1 | CATCCGAGTGAAACCCAAA (rev) |  |  |
|  | 5RmecA | TATACCAAACCCGACAACTAC (fwd) | *mecA–IS*431 (359) |  |
|  | 5R431 | CGGCTACAGTGATAACATCC (rev) |  |  |
| Virulence/  drug resistance genes | aac1 | CAGAGCCTTGGGAAGATGAAG (fwd) | *aac(6')-Ie-aph(2'')-Ia* | [5] |
|  | aac2 | CCTCGTGTAATTCATGTTCTGGC (rev) |  |  |
|  | ant1 | CAAACTGCTAAATCGGTAGAAGCC (fwd) | *aph(3')-IIIa* | [5] |
|  | ant2 | GGAAAGTTGACCAGACATTACGAACT (rev) |  |  |
|  | aph1 | AAGAATTTTTATCTTAATTAAGGAAGGAGTG (fwd) | *ant(4')-Ia* | [5] |
|  | aph2 | TTAGTGAATTTGTTCACTGTGTCGA (rev) |  |  |
|  | lukPV-1 | ATCATTAGGTAAAATGTCTGGACATGATCCA (fwd) | *PVL* (433) | [6] |
|  | lukPV-2 | GCATCAAATGTATTGGATAGCAAAAGC (rev) |  |  |
|  | mecA1 | GTAGAAATGACTGAACGTCCGATAA (fwd) | *mecA* (391) |  |
|  | mecA2 | CCAATTCCACATTGTTTCGGTCTAA (rev) |  |  |
|  | 486F | GTTGCGAACTCTTGAATAGG (fwd) | *blaZ* (674) | [7] |
|  | 486R | GGAGAATAAGCAACTATATCATC (rev) |  |  |
|  | icaSaF | TATGAACCGCTTGCCATGTG (fwd) | *ica***^2^** (854) | This study |
|  | icaSaR | GATGACCATCCAGTGTGCTTAC (rev) |  |  |
|  | sdrEF | AGTAAAATGTGTCAAAAGA (fwd) | *sdrE* (767) | [8] |
|  | sdrER | TTGACTACCAGGCTATAT (rev) |  |  |
|  | hlgF | GCCAATCCGTTATTAGAAAATGC (fwd) | *hlg* (937) |  |
|  | hlgR | CCATAGACGTAGCAACGGAT (rev) |  |  |
|  | cnaF | AGTGGTTACTAATACTG (fwd) | *cna* (744) |  |
|  | cnaR | CAGGATAGATTGGTTTA (rev) |  |  |
|  | hla1 | CTGATTACTATCCAAGAAATTCGATTG (fwd) | *hla* (209) | [9] |
|  | hla2 | CTTTCCAGCCTACTTTTTTATCAGT (rev) |  |  |
|  | hlb1 | GTGCACTTACTGACAATAGTGC (fwd) | *hlb* (309) |  |
|  | hlb2 | GTGCACTTACTGACAATAGTGC (rev) |  |  |
|  | hld1 | AAGAATTTTTATCTTAATTAAGGAAGGAGTG (fwd) | *hld* (111) |  |
|  | hld2 | TTAGTGAATTTGTTCACTGTGTCGA (rev) |  |  |
|  | tstF | ATGGCAGCATCAGCTTGATA (fwd) | *tst1* (350) | [10] |
|  | tstR | TTTCCAATAACCACCCGTTT (rev) |  |  |
|  | seAF | GGATATTGTTGATAAATATAAAGGGAAAAAAG (fwd) | *seA* (439) |  |
|  | seAR | GTTAATCGTTTTATTATCTCTATATATTCTTAATAGT (rev) |  |  |

**^1^**PCRs contained 20pmoles each of forward (fwd) and reverse (rev) primers, 1.5U *Taq* polymerase (Thermo Scientific, Surry, UK), Custom PCR-Master Mix (Thermo Scientific, Surry, UK), template DNA and nuclease-free water, in 10µl reaction volume. Amplification conditions are described in indicated citations.

**^2^**Primers icaSaF and icaSaR amplified 854bp of the *ica* operon containing *icaA*, *icaD* and *icaB* gene fragments under conditions, 94°C, 5 min; (94^o^C, 1 min, 60^o^C, 1 min &72^o^C, 1 min) x30 cycles; 72^o^C, 10 min. Primers were synthesized by IDT (Leuven, Belgium).

**Additional References**

1. Mignard S, Flandrois JP: **16S rRNA sequencing in routine bacterial identification: A 30-month experiment**. *Journal of Microbiological Methods* 2006, **67**(3):574-581.

2. Saha B, Singh AK, Ghosh A, Bal M: **Identification and characterization of a vancomycin-resistant Staphylococcus aureus isolated from Kolkata (South Asia)**. *J Med Microbiol* 2008, **57**(1):72-79.

3. Paule SM, Pasquariello AC, Hacek DM, Fisher AG, Thomson RB, Jr, Kaul KL, Peterson LR: **Direct Detection of Staphylococcus aureus from Adult and Neonate Nasal Swab Specimens Using Real-Time Polymerase Chain Reaction**. *J Mol Diagn* 2004, **6**(3):191-196.

4. Harmsen D, Claus H, Witte W, Rothganger J, Claus H, Turnwald D, Vogel U: **Typing of Methicillin-Resistant Staphylococcus aureus in a University Hospital Setting by Using Novel Software for spa Repeat Determination and Database Management**. *J Clin Microbiol* 2003, **41**(12):5442-5448.

5. Sundsfjord A, Simonsen GS, Haldorsen BC, Haaheim H, Hjelmevoll SO, Littauer P, Dahl KH: **Genetic methods for detection of antimicrobial resistance**. *APMIS* 2004, **112**(11-12):815-837.

6. McClure J-A, Conly JM, Lau V, Elsayed S, Louie T, Hutchins W, Zhang K: **Novel Multiplex PCR Assay for Detection of the Staphylococcal Virulence Marker Panton-Valentine Leukocidin Genes and Simultaneous Discrimination of Methicillin-Susceptible from -Resistant Staphylococci**. *J Clin Microbiol* 2006, **44**(3):1141-1144.

7. Olsen JE, Christensen H, Aarestrup FM: **Diversity and evolution of blaZ from Staphylococcus aureus and coagulase-negative staphylococci**. *Journal of Antimicrobial Chemotherapy* 2006, **57**(3):450-460.

8. Kumar JD, Negi YK, Gaur A, Khanna D: **Detection of virulence genes in Staphylococcus aureus isolated from paper currency**. *International Journal of Infectious Diseases* 2009, **13**(6):e450-e455.

9. Jarraud S, Mougel C, Thioulouse J, Lina G, Meugnier H, Forey F, Nesme X, Etienne J, Vandenesch F: **Relationships between Staphylococcus aureus Genetic Background, Virulence Factors, agr Groups (Alleles), and Human Disease**. *Infect Immun* 2002, **70**(2):631-641.

10. Stotts SN: **Virulence and Antibiotic Resistance Gene Combinations among Staphylococcus aureus Isolates from Coastal Waters of Oahu, Hawaii**. *The Journal of Young Investigators* 2005, **12**(4).
